# Quantifications of Outer Retinal Bands in Geographic Atrophy by Comparing Superior Axial Resolution and Conventional OCT

**DOI:** 10.1167/iovs.66.4.65

**Published:** 2025-04-22

**Authors:** Sophie Frank-Publig, Hrvoje Bogunovic, Klaudia Birner, Markus Gumpinger, Philipp Fuchs, Leonard M. Coulibaly, Virginia Mares, Friedrich Michel, Fiona Sophia Schmidt, Ursula Schmidt-Erfurth, Gregor S. Reiter

**Affiliations:** 1 Laboratory for Ophthalmic Image Analysis, Department of Ophthalmology and Optometry, Medical University of Vienna, Vienna, Austria; 2 Christian Doppler Laboratory for Artificial Intelligence in Retina, Department of Ophthalmology and Optometry, Medical University of Vienna, Vienna, Austria; 3 Institute of Artificial Intelligence, Center for Medical Data Science, Medical University of Vienna, Vienna, Austria

**Keywords:** age-related macular degeneration, geographic atrophy, optical coherence tomography, photoreceptors, artificial intelligence

## Abstract

**Purpose:**

Novel treatments for geographic atrophy (GA) require precise monitoring, which can be improved with advances in optical coherence tomography (OCT) technology. The purpose of this study was to investigate the benefits of a novel device with superior axial resolution.

**Methods:**

Patients were recruited at the Department of Ophthalmology and Optometry at the Medical University of Vienna. Patients with GA were imaged with a Heidelberg SPECTRALIS HRA+OCT and the novel Heidelberg High-Res OCT device. Outer retinal bands and subretinal drusenoid deposits (SDDs) were segmented in 49 B-scans per OCT. Thickness and loss of outer retinal bands, as well as SDD volumes, were compared between devices and regions using linear mixed-effects models.

**Results:**

The study included 3920 B-scans of 40 eyes of 32 patients. For the High-Res OCT, the myoid zone was thinner (19.85 µm, 95% confidence interval [CI] 16.8–22.8 vs. 21.37 µm, 95% CI 18.4–24.4; *P* < 0.001), whereas the ellipsoid zone (EZ) band was thicker (28.35 µm; 95% CI 22.7–24.0 vs. 27.29 µm, 95% CI 21.6–33.0). Smaller EZ- and external limiting membrane loss areas (all *P* < 0.001) were found for the High-Res OCT. The RPE band was thinner for the High-Res OCT (15.97 µm, 95% CI 13.5–18.4 vs. 21.08 µm, 95% CI 18.6–23.5; *P* < 0.001) without significant differences in RPE loss. Higher SDD volumes were found for the High-Res OCT (*P* < 0.001).

**Conclusions:**

Precise in vivo quantification of OCT features is of great relevance for individualized patient management. The High-Res OCT device allows for detailed topographical analysis of outer retinal changes in GA, which could improve early detection, patient selection, and patient management in clinical practice.

Age-related macular degeneration (AMD) including geographic atrophy (GA) is a substantial burden for millions of individuals worldwide.^1^ Novel therapeutic developments (e.g., complement inhibition) hold promise for revolutionizing GA management by decelerating the speed of atrophy progression. Visual acuity has been shown to be a weak endpoint for measuring the efficiency of these new therapies; therefore, anatomical endpoints have been approved by regulatory authorities for clinical trials. Optical coherence tomography (OCT) is the preferred imaging modality due to the impact of the disease beyond the retinal pigment epithelium (RPE) to the photoreceptors (PRs).^2^ Early OCT technologies imaged the outer retina as one thick hyperreflective line by combining signals from adjoining bands.^3^^,^^4^ With improvements in speed and resolution, more details became visible and terminology had to be adjusted repeatedly.^4^ Today, spectral-domain OCT (SD-OCT) achieves fast, high-resolution visualization of ultrastructural changes in the retina. In OCT B-scans, the outer retina is visualized as four hyperreflective lines: band 1, external limiting membrane (ELM); band 2, ellipsoid zone (EZ); band 3, interdigitation zone (IZ); and band 4, RPE/Bruch's membrane (BM) complex.^4^^–^^6^ In ultrahigh-resolution OCT, the hyperreflective band posterior to the ELM represents the ellipsoid zone of the inner segment of the PR. Below the EZ lie the cone outer segment tips (COSTs), rod outer segment tips (ROSTs), RPE, and BM. The positioning of ROSTs posterior to COSTs has been described by adaptive optics OCT, as well.^7^^–^^9^

However, for precise quantification of retinal layers, drusen can cause disturbed waveguiding properties resulting in attenuated and distorted signals in adaptive optics OCT-based PR analysis, suggesting integrity loss. Furthermore, displaced OCT scans with an oblique angle of incidence can artificially thicken retinal bands.^10^ Previous research correlating histology to outer hyperreflective bands in OCT found that bands 1 to 4 contain the junctions of Müller cells, the inner and outer segments of PRs and RPE.^4^^,^^5^ Nevertheless, histologic sections are not ideal for quantification of retinal layers. The processing of tissues in histologic analyses causes tissue volume changes, retinal detachments, and thin sections.^11^ On the other hand, OCT-based measurements tend to overestimate the thickness of outer retinal layers due to blurred reflections and contrast adjustments. These modifications can also artificially increase the distance between the RPE and the IZ bands.^4^^,^^5^ Although histology is of utmost importance in the validation of in vivo imaging, the question remains unanswered as to which method is best suited for quantitative measurements of the retinal morphology.

Although conventional SD-OCT has been successful in reliably imaging retinal structures in GA,^12^^,^^13^ superior axial resolution OCT appears to introduce further benefits.^14^ A novel investigational OCT device, the High-Res OCT, achieves an axial resolution of 3 µm compared to 7 µm in the SPECTRALIS HRA+OCT (both Heidelberg Engineering, Heidelberg, Germany). Improvements in inter- and intra-reader reliability for RPE, EZ, and ELM annotations, as well as hypertransmission, suggest superiority in retinal visualization.^15^ Similarly, another group reported higher accuracy in identifying incomplete RPE and outer retinal atrophy (iRORA) in addition to the superiority of the High-Res OCT in qualitative and quantitative assessment of RPE loss, EZ loss, and ELM loss in AMD.^16^ This study aimed to present quantitative measurements in predefined subfields for both devices individually to provide reliable and precise reference points for future studies and to compare these measurements between devices and subfields. Furthermore, we aimed to describe disease-characteristic morphologies in high-resolution OCT imaging.

## Methods

This study was approved by the ethics committee of the Medical University of Vienna and adhered to the tenets of the Declaration of Helsinki. All patients involved provided written consent prior to imaging. Recruitment took place from September 2021 to June 2023 at the outpatient clinic of the Department of Ophthalmology and Optometry at the Medical University of Vienna. In this cross-sectional study, patient eligibility was confirmed on OCT imaging with complete RPE and outer retinal atrophy (cRORA) as defined previously.^17^ Patients with signs of neovascular AMD and OCT images of low quality were excluded. Exclusion criteria for low quality were (1) mirroring of outer retinal bands in one or more B-scans, (2) capping of the outer retinal bands in one or more B-scans, (3) blurred images beyond the feasibility of reliable segmentation due to lacking fixation or dry eyes, and (4) decentered images, where the fovea was located outside the central 1-mm circle of the OCT volume. Most of these artifacts derived from problematic fixation in patients with fovea-involving GA, which was the case for both devices. Furthermore, we included only eyes with <6 diopter (D) spherical aberration to account for biased results or inferior image quality due to overly short or long eyes. Additionally, to account for an impact of varying eye lengths in participants, we calculated the spherical equivalent from medical records of the visit closest to the image acquisition.

### Devices and Image Acquisition

The investigational High-Res OCT uses a super luminescent diode-based light source that provides a larger spectral bandwidth of 137 nm compared to 50 nm in the standard SPECTRALIS HRA+OCT next to a shorter central wavelength (850 nm vs. 880 nm), resulting in up to 3-µm axial resolution, whereas conventional OCT reaches 7 µm. The lateral resolution remains identical, but the presumably higher power at the pupil of 2.2 mW compared to 1.2 mW in standard SPECTRALIS OCT improves the signal-to-noise ratio, which can enhance the contrast. Patients were imaged on two devices, standard SPECTRALIS HRA+OCT and the High-Res OCT at the same visit using the follow-up function by exporting the first OCT on the standard device and importing it in a masked fashion on the High-Res OCT device, allowing for superimposed images. Study procedures included the acquisition of fovea-centered 20° × 20° cubes in high-resolution mode with automated real time set to 16. All OCTs acquired had 1024 A-scans and 97 B-scans except two OCT volumes deriving from one patient, which had only 49 B-scans. These two acquisitions deviated from the 97 B-scan protocol due to a long-term follow-up with 49 B-scans as a result of a lack of concentration in this patient. However, due to the limited number of eyes included in the study and the identical number of A-scans, we chose to include and compare the images. Furthermore, quantitative measurements were only calculated in 49 manually corrected B-scans, because previous research has suggested no significant differences in quantification of retinal features in AMD between 49 and 97 B-scans.^18^ If the fovea was not automatically detected correctly, the foveal center point was set manually before the first acquisition. Patients received 0.5% tropicamide and artificial tears for optimal imaging quality.

### Outer Retinal Band Segmentation and Quantification

**Figure 1.**
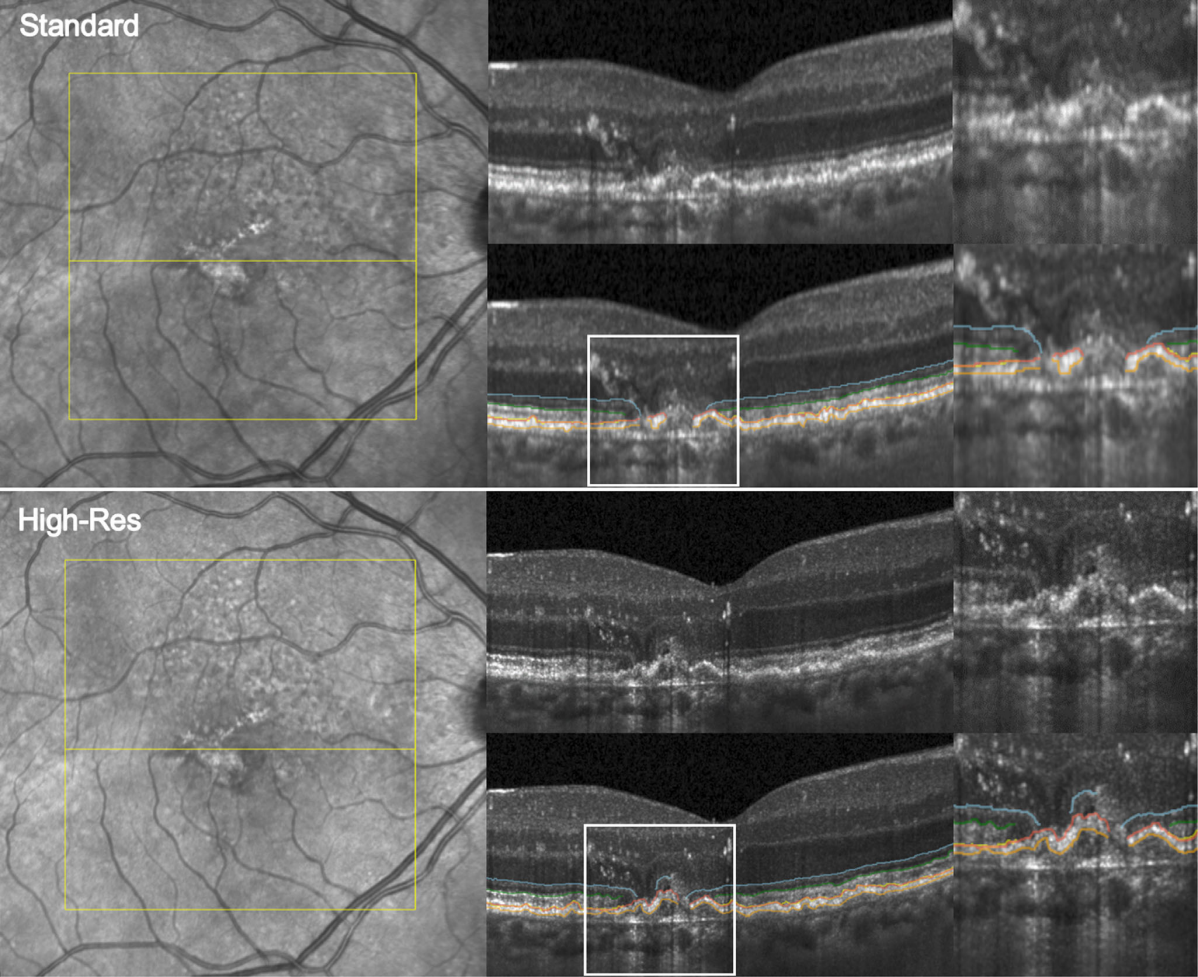
Eighty-year-old patient with geographic atrophy. (*Top row*) En face scanning laser ophthalmoscopy (SLO) and OCT B-scans (*yellow line* on SLO) acquired with SPECTRALIS HRA+OCT. (*Bottom row*) En face SLO and OCT B-scans (*yellow line*) acquired with the High-Res OCT device. The B-scans show layer segmentation of the outer (*orange*) and inner (*red*) RPE borders, the outer (*yellow*) and inner (*white*) border of the EZ, and the ELM (*blue*). In the *right column*, the area of the *white square* from the OCT B-scans is magnified.

In this study, outer retinal band quantifications include the RPE, EZ, myoid zone (MZ), and ELM. First, automated artificial intelligence (AI) algorithms presegmented the RPE and EZ bands to generate the necessary segmentation lines. All segmentation lines were then manually corrected in 49 B-scans per OCT volume with regular intervals of 122 µm (Fig. 1). The inner and outer boundaries of the RPE band were presegmented using the Iowa Reference Algorithm (Retinal Image Analysis Lab, Iowa Institute for Biomedical Imaging, Iowa City, IA, USA).^19^ The RPE band was defined as the anterior portion of the 14th zone based on the international nomenclature for OCT, also known as the fourth outermost hyperreflective band of the outer retina.^20^ In some sections of high-resolution OCT, this band can be visible as two hyperreflective lines separated by a hyporeflective zone, particularly in regions with basal laminar deposits (BLamDs) or drusen. We segmented the outer boundary of the anterior hyperreflective band as the outer boundary of the RPE band excluding BLamDs, drusen, or BM, when visible. RPE loss was defined as disruption of the RPE band, and the segmentation lines were cut where the hyperreflectivity ended. RPE loss was calculated from the resulting cut out area and indicated in square millimeters. RPE migrations (sloughed RPE)^21^ separated from the RPE band by a hyporeflective zone, therefore, located in the subretinal space were included in EZ thickness measurements. Basal shedding of granule aggregates visible as hyperreflective material separated by hyporeflectivity posterior to the RPE band were also not included in RPE thickness measurements. Between the inner boundary of the RPE and the outer boundary of the EZ band, subretinal drusenoid deposits (SDDs) were manually segmented and defined as mound-shaped supplemental granular hyperreflective material in the IZ.^22^^,^^23^ Previous inter-reader agreement assessments reported limited reliability for stage 1 SDDs; therefore, they were not included in our SDD quantifications.^24^ SDD volume was registered over a threshold of 4 µm (= 1 pixel) to account for human error in layer segmentation. The inner and outer boundaries of the EZ band were presegmented using a deep-learning algorithm based on an ensemble U-net.^25^ In this study, EZ band measurements included the IZ and the EZ,^6^ whereas, outside of the fovea, two hyperreflective lines become visible posterior to the EZ. As only cone PRs are located in the fovea and rod density increases with eccentricity, we suggest that these lines represent ROSTs and COSTs separated by a hyporeflective zone. The outer boundary of the EZ band was drawn posterior to these lines at the inner boundary of RPE and above the SDDs. If SDDs visibly disrupted the EZ or ELM, these A-scan regions were marked as EZ loss due to evidence of displacement of the EZ.^26^ Between the ELM and the inner boundary of the EZ lies the myoid zone, which was included in thickness quantifications. The ELM+MZ thickness in this study consists of the ELM and the MZ. The foveal center, defined as the central A-scan, where inner retinal bands merge together closest on the B-scan with the maximum foveal depression, was manually annotated by an expert reader (SF) in each OCT volume to account for problematic detection and fixation in GA patients.

Overall, five segmentation lines were manually corrected in 49 B-scans per OCT volume by one of seven expert readers in the first round, and each B-scan was re-corrected when necessary by a senior expert reader (SF) in a second round for consistency. The readers were provided a reading manual by the senior reader (SF), and meetings for training and supervision took place weekly. All annotations were conducted using the in-house–developed annotation tool OPTIMUS 1.8.6.90 (Medical University of Vienna, Vienna, Austria).

### Statistical Analysis

Band thickness and loss were calculated from manually corrected OCT B-scans for the overall field of view, as well as for topographic regions of the Early Treatment Diabetic Retinopathy Study (EDTRS) grid centered on the manually annotated foveal center point. Quantifications of loss areas were normalized to the central, parafoveal (1–3 mm), and perifoveal (3–6 mm) ring area sizes (0.79 mm^2^, 6.28 mm^2^, and 21.21 mm^2^, respectively) indicated as absolute (mm^2^) and relative loss per mm^2^ (%) measurements for the analysis of those regions. For topographic SDD analysis, absolute volumes of summarized superior, nasal, inferior, and temporal quadrants of the parafoveal and perifoveal subfields of the EDTRS grid were compared between the central 1-mm circle and the respective quadrants, as well as between devices. Comparisons between devices and regions were conducted using mixed-effects models while accounting for inclusion of both eyes for some patients. For multiple testing, *P* values were corrected using Bonferroni correction. In the device comparison, logarithmic transformation was performed for loss areas in the entire 20° × 20° OCT volume due to skewed data distribution. For all metric measurements, mean and 95% confidence intervals (CIs) were indicated, and log-transformed data were reported after back-transformation. A *P* value was considered significant below α = 0.05.

## Results

**Figure 2.**
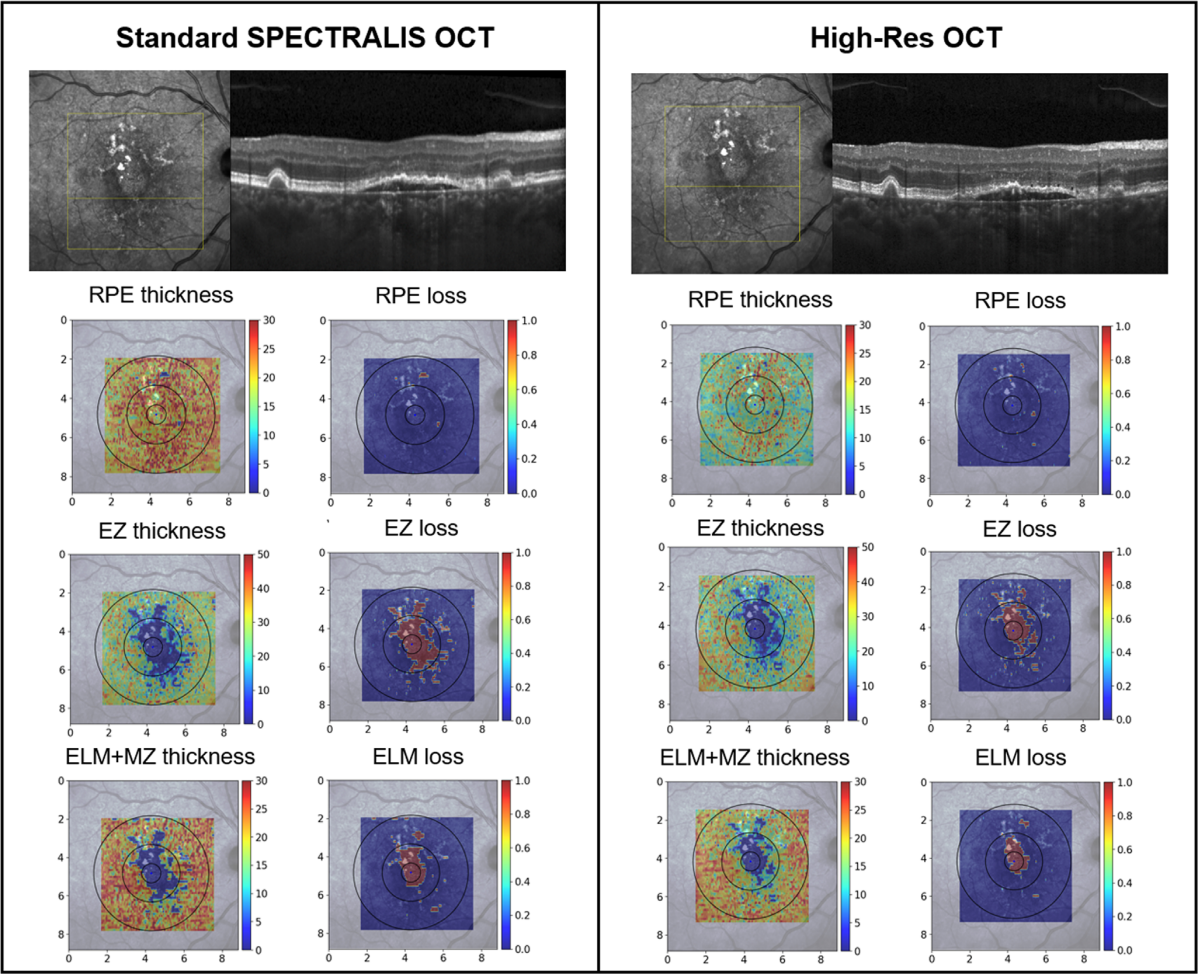
Topographic maps of a 77-year-old patient with geographic atrophy. Layer thickness (µm) maps are presented in the *left column*, and the *right column* shows differences in loss (mm^2^) of the reflective bands under investigation. The panels indicating RPE thickness suggest overall thinner layers in the High-Res OCT with similar losses. For EZ thickness, particular inferior–nasal to the fovea, higher losses and overall thicker layers are visible in the High-Res OCT on the *right*. A large difference between devices can be seen in ELM loss.

Forty eyes from 32 patients with GA secondary to AMD were included; 18 eyes (45%) were right eyes, 18 patients (56%) were female, and mean age was 78.9 ± 5.4 years. The mean spherical equivalent for all eyes was −0.08 ± 0.99 D. Overall, five lines were manually corrected twice in 3920 B-scans. Based on these measurements, topographic maps of SDDs, thickness, and loss of outer retinal bands were computed for each eye (Fig. 2).

### Device-Associated Differences

**Figure 3.**
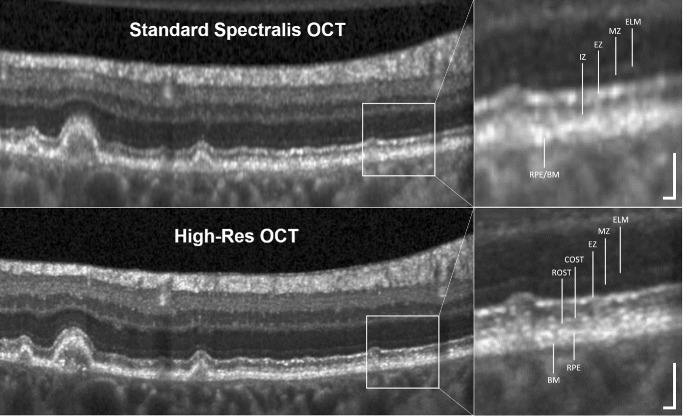
A 74-year-old patient with geographic atrophy. Differences in outer hyperreflective bands in standard SPECTRALIS HRA+OCT and the High-Res OCT can be seen. In the *top* *row*, we can differentiate hyperreflective bands of the outer retina (from *bottom* to *top*) into the BM/RPE complex, the IZ, the EZ, the hyporeflective MZ, and the ELM as described by Staurenghi et al.^6^ In the *bottom row*, images from the High-Res OCT revealed additional hyporeflective zones separating BM from the RPE, and outside of the fovea two lines become visible. These lines have been correlated to ROSTs and COSTs.^7^^,^^8^ The *bars* in the *righthand corner* of the magnification panels measure 50 µm vertically and horizontally. The signal-to-noise ratio for the standard SPECTRALIS OCT was 1.14 (0.57 dB), and it was 1.15 (0.61 dB) for the High-Res OCT.

Improved axial resolution resulted in a clearer distinction between the outer retinal bands in OCT imaging (Fig. 3). All measurements of the entire OCT volume regarding differences between standard SPECTRALIS HRA+OCT and the High-Res OCT are presented in Table 1. For thickness measurements in the entire OCT volume (20° × 20°), the ELM+MZ thickness was significantly thinner on the High-Res OCT (*P* < 0.001). Furthermore, due to improved delineation of the RPE boundaries, the EZ band was thicker in the High-Res OCT (*P* < 0.001), and RPE measurements showed a thinner band compared to standard SPECTRALIS OCT (*P* < 0.001). For the comparison of integrity loss between devices, loss of outer retinal bands was determined for the entire OCT volume (20° × 20°). Areas with intact EZ and ELM integrity were larger in the High-Res OCT compared to standard SPECTRALIS HRA+OCT (both *P* < 0.001). No significant differences between devices were found for the area of RPE loss (*P* = 0.259). Finally, significantly higher SDD volumes were found in the High-Res OCT device compared to standard SPECTRALIS HRA+OCT (Table 1).

**Table 1.** Outer Retinal Band Quantifications of the Entire OCT Volume (20° × 20° Acquisition) for Standard SPECTRALIS HRA+OCT and the High-Res OCT With 7-µm and 3-µm Axial Resolution

	Standard SPECTRALIS HRA+OCT		High-Res OCT		
	Mean	95% CI	Mean	95% CI	*P*
ELM+MZ thickness (µm)	21.37	18.36–24.38	19.85	16.84–22.86	<0.001
EZ thickness (µm)	27.29	21.63–32.96	28.35	22.68–34.02	<0.001
RPE thickness (µm)	21.08	18.63–23.53	15.97	13.52–18.42	<0.001
ELM loss (mm^2^)^*^	2.48	0.28–21.93	1.96	0.22–17.34	<0.001
EZ loss (mm^2^)^*^	4.08	0.67–24.72	3.35	0.55–20.28	<0.001
RPE loss (mm^2^)^*^	1.57	0.15–16.33	1.66	0.16–17.30	0.259
SDD volume (nL)	78.42	50.26–106.59	135.94	107.78–164.10	<0.001

CI, Confidence interval; ELM, external limiting membrane; EZ, ellipsoid zone; RPE, retinal pigment epithelium; SDD, Subretinal drusenoid deposits.

Thicknesses and layer losses were compared between devices using a mixed-effects model.

* Calculated using log-transformed data in a mixed-effects model due to right-skewed distribution. Mean and 95% CI are reported after back-transformation.

### Topographic Differences in Superior Axial Resolution

**Figure 4.**
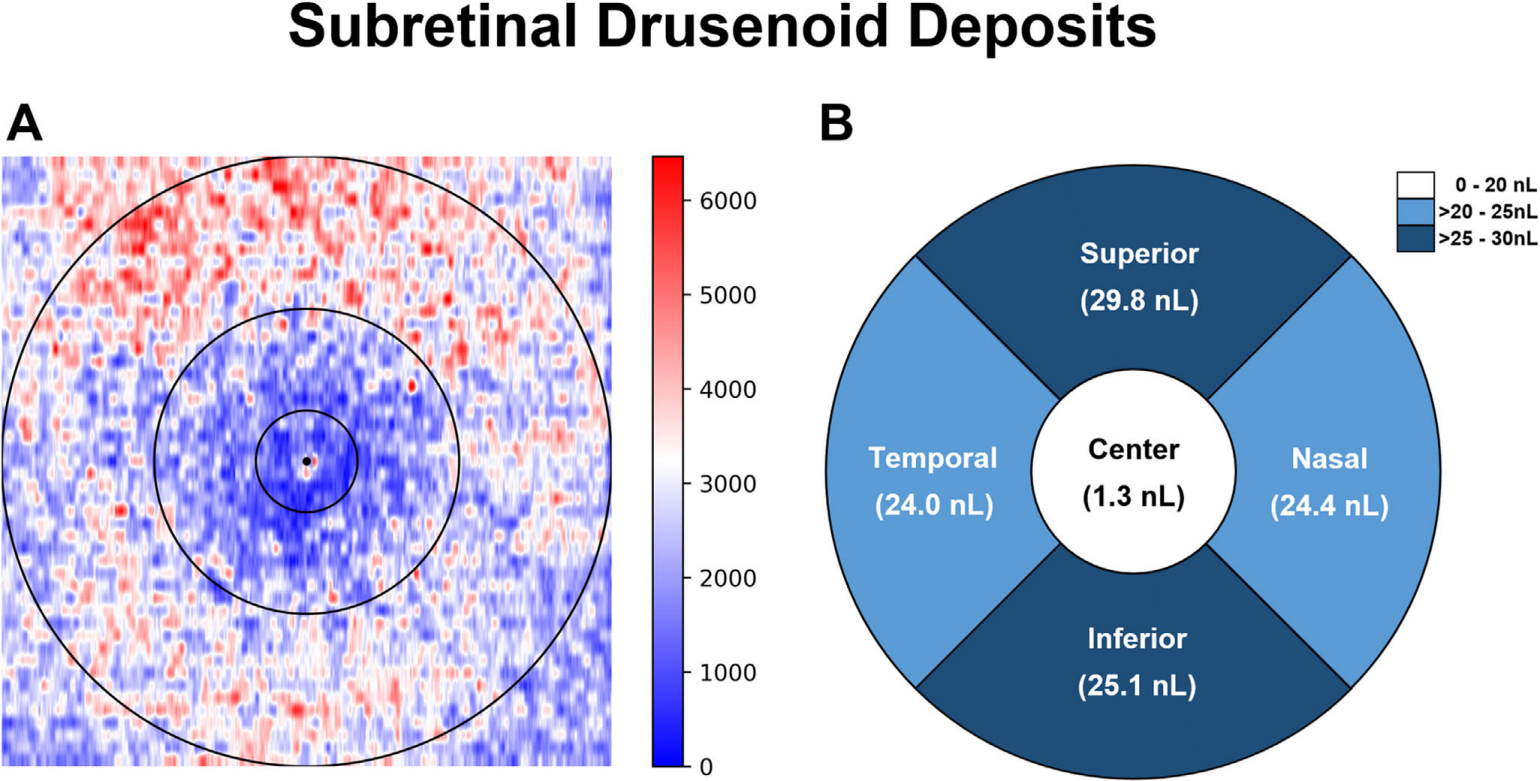
Schematic illustration of SDD volume distribution. (**A**) A summarized map of SDD volumes was calculated as the average en face projected SDD volume per pixel in cubic micrometers. It demonstrates lower volumes in the central 1 mm with higher accumulation in the para- and perifoveal rings. (**B**) The topographical distribution of SDD volumes is illustrated. Mean values are indicated in parentheses below the respective subfields. A mixed-effects model was calculated to compare those regions while accounting for inclusion of both eyes for some patients and correcting for multiple testing using Bonferroni correction.

The topographic analysis of these retinal features calculated the loss and thickness of outer retinal bands in foveal-centered regions of the EDTRS grid, including the central 1 mm, parafoveal 1- to 3-mm ring, and perifoveal 3- to 6-mm ring. Table 2 shows all measurements for both devices for reference in future studies assessing the OCT-based quantifications of outer retinal bands in GA. Mean and 95% CIs for all parameters were compared for the High-Res OCT, as retinal layers can be visualized in greater detail due to superior axial resolution and higher power. Mean EZ thicknesses were 20.43 µm, 23.92 µm, and 27.96 µm in the central, parafoveal, and perifoveal rings, respectively, indicating a thinner layer with closer proximity to the fovea (all *P* < 0.05). RPE and ELM+MZ thicknesses remained similar across all regions. For RPE integrity, the central and parafoveal regions contained higher losses than the perifoveal region (both *P* < 0.01). Similarly, a smaller area of EZ loss was found in the perifoveal region (both *P* < 0.001) with a tendency of highest losses in the central area (*P* = 0.059). Equally, ELM loss was lowest in the perifoveal area (both *P* < 0.01) with a tendency toward maximum loss in the central subfield (*P* = 0.063). In conclusion, highest losses of the RPE, EZ, and ELM bands were found in the central and parafoveal regions. For SDDs, the lowest volume was found in the central subfield (all *P* < 0.001) with the highest volume superiorly (Table 3); however, there were no significant differences among the superior, nasal, inferior, and temporal quadrants (all *P* < 0.05) (Fig. 4). When comparing SDD volumes normalized to area size, only the nasal subfield contained lower volumes than the temporal subfield (*P* = 0.01).

**Table 2.** Layer Quantifications in Predefined EDTRS Fields Including the Center 1 mm, Parafoveal Ring Around the Center (1–3 mm), and Perifoveal Ring (3–6 mm)

	Topographic Measurements of Outer Retinal Bands					
	Center		Parafoveal Ring		Perifoveal Ring	
Layer	Mean (rel)	95% CI	Mean (rel)	95% CI	Mean (rel)	95% CI
Standard SPECTRALIS HRA+OCT						
ELM+MZ thickness (µm)	18.14	15.9–20.4	21.27	19.1–23.5	20.82	18.6–23.0
EZ thickness (µm)	19.33	16.6–22.0	27.02	24.3–29.7	23.54	20.8–26.3
RPE thickness (µm)	20.02	18.5–21.5	21.16	19.7–22.7	21.56	20.1–23.1
ELM loss (mm^2^)^*^	0.34 (43.3%)	25.8–60.9	3.38 (53.7%)	36.2–71.3	2.23 (10.5%)	−7.0 to 28.1
EZ loss (mm^2^)^*^	0.42 (53.8%)	34.5–73.2	4.16 (66.2%)	46.9–85.5	2.84 (13.4%)	−5.9 to 32.7
RPE loss (mm^2^)^*^	0.30 (37.7%)	21.7–53.7	2.73 (43.4%)	27.3–59.4	1.97 (9.3%)	−6.8 to 25.3
High-Res OCT						
ELM+MZ thickness (µm)	18.94	9.4–28.5	19.40	9.8–29.0	19.79	10.2–29.4
EZ thickness (µm)	20.43	8.7–32.2	23.92	12.2–35.7	27.96	16.2–39.7
RPE thickness (µm)	15.71	10.0–21.5	17.36	11.6–23.1	16.04	10.3–21.8
ELM loss (mm^2^)^*^	0.33 (42.5%)	−7.3 to 92.2	2.21 (32.8%)	0.3–19.5	3.01 (14.2%)	−35.6 to 63.9
EZ loss (mm^2^)^*^	0.40 (50.7%)	0.4–100.9	2.55 (40.6%)	−9.7 to 90.8	3.77 (17.8%)	−32.5 to 68.1
RPE loss (mm^2^)^*^	0.30 (38.4%)	−9.4–86.2	1.93 (30.7%)	−17.1 to 78.5	2.65 (12.5%)	−35.3 to 60.4

EDTRS, Early Treatment Diabetic Retinopathy Study.

* Quantifications of loss areas were normalized to the area of the respective EDTRS field (0.79 mm^2^, 6.28 mm^2^ and 21.21 mm^2^, respectively) and indicated as absolute measurements in mm^2^ and as relative loss per mm^2^ (rel) in %.

**Table 3.** Volumetric Quantification of SDDs

	Standard SPECTRALIS HRA+OCT		High-Res OCT	
	Mean (nL)	95% CI	Mean (nL)	95% CI
Center	0.66	−3.2 to 4.5	1.27	−4.9 to 7.5
Superior	16.46	12.6–20.3	29.79	23.6–36.0
Nasal	12.30	8.4–16.2	24.37	18.2–30.6
Inferior	12.59	8.7–16.4	25.09	18.9–31.3
Temporal	13.18	9.3–17.0	24.06	17.9–30.3

Absolute SDD volume was measured for the central 1 mm and for superior, nasal, inferior, and temporal parafoveal and perifoveal subfields of the EDTRS grid. Comparisons between the respective subfields showed significantly lower SDD volumes in the central 1-mm area compared to each quadrant. No differences were found among the superior, nasal, inferior, and temporal quadrants.

## Discussion

The High-Res OCT device enables precise visualization of outer retinal layers, which play a key role in the pathogenesis and progression of GA.^2^^,^^27^^,^^28^ In this study, quantitative measurements of outer retinal band thickness and loss from in vivo imaging differed significantly between two devices with differing axial resolution. OCT provides three-dimensional in vivo images of the retina, which is a requirement for quantification of retinal layers. Due to improvements in resolution and speed, one thick outer hyperreflective band^3^ could be converted to separable bands. In the High-Res OCT, the outer retinal band patterns in this study were in accordance with the literature and corresponded to the ELM, MZ, EZ, IZ, RPE, and BM.^4^^–^^6^^,^^29^ However, the space between the EZ and RPE bands revealed two hyperreflective bands outside of the fovea, presumably corresponding to ROSTs posteriorly to the COSTs^7^ (Fig. 3). This assumption is supported by ultrahigh-resolution OCT^7^^,^^30^^,^^31^ and adaptive optics OCT.^8^^,^^9^^,^^32^^,^^33^ The validated reflectivity model–based (RefMoB) method of Gaussian functions analyzing hyperreflective patterns on SPECTRALIS OCT vertically is a precise and reliable technique for quantification of hyperreflective bands in the outer retina.^5^ Due to limited research in OCT-based thickness measurements of outer retinal bands in GA, we compared our results to healthy eyes, as well.

Other OCT-based analyses have demonstrated 23.1 to 32.9 µm of MZ thickness.^4^^,^^5^^,^^7^ However, our results extended from 18.14 µm to 21.37 µm in eyes with GA, presumably due to degenerative shrinking of the myoid zone of the PR inner segment due to mitochondrial translocation toward the nucleus of the PR cells.^27^^,^^34^ Simultaneously, in GA the MZ thins with closer proximity to the border of the atrophy,^27^ which could also have a thinning impact on MZ thickness measurements in GA compared to normal eyes.

For EZ band thickness, the segmentation lines chosen vary across the literature, frequently measuring from the inner boundary of the outer nuclear layer to the RPE or solely the EZ. However, the EZ band under investigation in this study was between the inner boundaries of the RPE and EZ, in accordance with Riedl et al.^2^ for several reasons. First, there is a close connection between the PR inner segments and the PR outer segments located in the IZ.^20^ Second, other studies have included the outer nuclear layer in outer retinal layer segmentations^35^ because it contains the nucleus of the PR.^27^ However, the outer nuclear layer would not be a suitable candidate as a PR boundary in GA because of an unclear inner boundary; also, thickened outer nuclear layers in atrophic areas frequently contain almost exclusively Müller glia without viable PR.^27^ Therefore, measuring the EZ band as a combination of IZ and EZ is a better indicator of PR integrity. Third, structure–function correlations showed a strong correlation between EZ integrity and retinal function in early and late AMD.^36^^,^^37^ Furthermore, the mitochondria-packed EZ is representative of the metabolic health of the outer retina.^4^^,^^34^ Finally, reduced reflectivity of the EZ has been associated with faster disease progression.^38^ The U.S. Food and Drug Administration has recognized automated quantification of EZ attenuation as a primary endpoint in GA trials.^39^ In conclusion, the investigated outer retinal bands in this study contained the most viable PR structures for function and served as reliable segmentation lines.

RefMoB-based measurements demonstrated a thickening of the EZ toward the foveal center in normal eyes.^4^^,^^5^ Such a central thickening could not be confirmed in our study, where topographic comparisons showed thicker measurements toward the perifovea, presumably due to EZ thinning and higher losses in the central and parafoveal regions in GA. Furthermore, our EZ band was thicker than EZ measurements alone, because it was measured as a combination of the IZ and EZ. Nevertheless, in the High-Res OCT, the EZ was thicker and RPE thinner due to improved demarcation of the IZ toward the RPE band.

**Figure 5.**
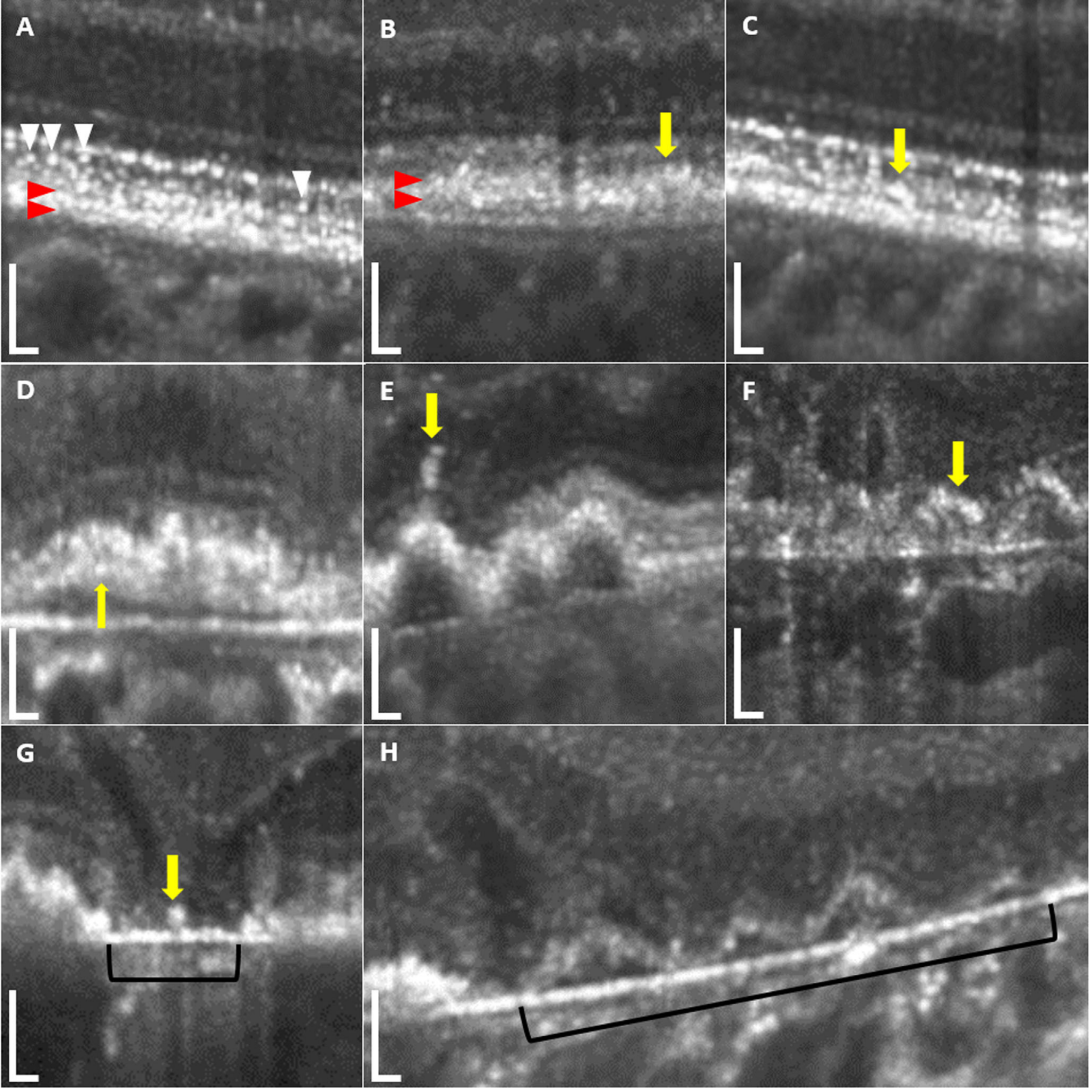
AMD-characteristic RPE phenotypes in the High-Res OCT as defined by the Project MACULA consortium and referred to in OCT by Zanzottera et al.^21^ (**A**) Nonuniform RPE, with a clearly delineated hyperreflective RPE layer (*red arrowheads*) anterior to BM. *White arrowheads* indicate subretinal hyperreflective spots defined as small hyperreflective circles located between the inner boundary of the IZ and the outer boundary of the EZ bands without hyporeflective tail. (**B**) Very nonuniform RPE, with an irregular hyperreflective RPE layer without clear borders. The *yellow arrow* points to shedding RPE adding to the underlying basal laminar deposits with intact epithelium above (OCT reference for very nonuniform in Fig. 5A^54^ and shedding RPE in Figs. 4 and 5,^12^). (**C**) Sloughed RPE. The *yellow arrow* shows a spherical hyperreflectivity similar to the underlying RPE in the subretinal space as referred to in figure 4 of Fleckenstein et al.^12^ (**D**) Bilaminar RPE. A hyperreflective layer formation is visible next to the RPE layer (*yellow arrow*). (**E**) Intraretinal RPE, with spherical hyperreflectivity similar to the underlying RPE anterior of the ELM (SD-OCT reference Fig. 4^12^). (**F**) Dissociated RPE. The *yellow arrow* points to a layer formation in an atrophic area with overlying ELM loss (reference figures 5, 6, and 7 of Fleckenstein et al.^12^). The structure shows similar hyperreflectivity as the RPE at the border of the atrophy with a small break in hypertransmission, also referred to as barcode’ stripes. (**G**) Atrophy without basal laminar deposits. Other than a dissociated RPE cell (*yellow arrow*), no hyperreflective remnants are visible on top of BM in the atrophic area indicated by the black bracket. (**H**) Atrophy with basal laminar deposits. Basal laminar deposits in this image are visible as a hyperreflective layer in the atrophic region indicated by RPE loss as described in Li et al.^27^ It is visible as a wavy constellation (*black brackets*) with overlying outer plexiform layer subsidence and underlying hypertransmission. SD-OCT references for atrophy with and without basal laminar deposits are figures 7C, 7F, 7G, and 7A of Fleckenstein et al.,^12^ respectively. Vacuolated and entombed RPE could not be identified in the investigated High-Res OCT images. *Scale bar*: 100 µm vertically and horizontally.

To interpret thickness and loss in diseased RPE, one should first understand the multiple alterations in pathogenetic morphology. In GA, the RPE continuously degenerates, and two main pathways of this process have been described: First, spherical RPE cells migrate into the subretinal space (referred to as sloughing) and further anteriorly into the inner retina (intraretinal). The second pathway is characterized by shedding of granules into the sub-RPE space adding material to underlying BLamDs as in apoptosis.^28^^,^^40^^,^^41^ Thereafter, pigmented RPE cells detach from the layer and are scattered across areas of atrophy (dissociated),^28^ so the borders of atrophy are not clearly demarcated by RPE loss primarily due to residual RPE (fragments) and BLamDs in atrophic areas.^27^ Recognizing multiple stress responses of RPE in vivo in OCT imaging could be beneficial in the interpretation of disease progression and treatment response in the future.^42^^–^^45^ These RPE phenotypes in GA have been further defined in a grading system based on histologic sections and OCT by the Project MACULA consortium, which assigned published images to specific phenotypes of RPE on OCT, as well.^21^ In Figure 5, correlates of some RPE phenotypes based on these descriptions are demonstrated for the High-Res OCT. In this study, the High-Res OCT demonstrated a detailed visualization of the RPE phenotypes due to superior axial resolution and higher power improving contrast, potentially enabling further distinctions between relevant RPE islands such as foveal-sparing, dissociated cells or BLamDs. For the High-Res OCT, a fine hyporeflective line separated the RPE band from the adjoining BM and IZ in more sections compared to the standard device due to higher axial resolution, which explains the significantly thinner measurements of this monolayer. Simultaneously, an improved delineation of BLamDs and drusen posterior to the RPE in the High-Res OCT could explain the device-dependent difference in thickness measurements.

There are some studies assessing RPE band thickness on OCT in normal eyes; however, limited results are available for GA eyes, presumably due to difficulties in the segmentation of the irregular morphology of the RPE in GA. RefMoB-based measurements in normal eyes found a thinner RPE band compared to results from this study.^5^ The previously described RPE irregularities could be the cause for thicker measurements in GA compared to normal eyes, as well as BLamDs posterior to the RPE. Furthermore, a thicker RPE layer at the borders of GA has been reported.^28^^,^^46^ Nevertheless, in our study, the RPE band measurements in the High-Res OCT were closer to measurements using the RefMob technique, which analyzes hyperreflective patterns on OCT with gaussian curves, demonstrating detailed visualization of outer retinal bands due to higher axial resolution.

Histologic studies are the basis of understanding retinal morphology and disease patterns in GA; therefore, we aimed to compare our results to findings from histologic sections. Thickness measurements of outer retinal layers from histologic sections are rare in GA eyes, so we compared only measurements of the RPE layer. RPE thickness measurements vary across histologic analyses. Evaluations of RPE thickness in histologic sections of eyes with GA have shown that “nonuniform” RPE spanned 11.7 ± 2.6 µm, but other phenotypes were found to be thicker, with vacuolated RPE measuring up to 19.5 µm and sloughed RPE up to 17.0 µm.^21^ Furthermore, other characteristic morphologic changes can be present that would lead to more variety across measurements, such as BLamDs. A previous study showed that RPE without BLamDs was 12.1 µm thick, but when combined with BLamDs the layer spanned 19.3 µm.^47^ Another study reported a combined thickness of BLamDs and RPE from histologic sections of 17.4 µm in eyes with GA compared to 12.7 µm for RPE alone. They concluded that a thickened RPE and BLamD layer combined compared to normal eyes resulted from expansion of BLamDs in AMD.^47^ However, despite differences across measurements, the layer thickens with closer proximity to the atrophic border in GA.^27^ Our results suggest a thicker RPE layer than in histologic sections of eyes with GA and normal eyes in OCT, which could be explained by (1) tissue shrinkage in histologic sections,^11^ (2) the inclusion of non-epithelial RPE phenotypes, and (3) thickening of the RPE layer close to atrophy.^28^^,^^46^ However, our topographic comparison demonstrated higher layer loss in the central and parafoveal regions without thickening of the RPE layer. Our measurements of the RPE band were 21 µm for standard OCT and 16 µm for the High-Res OCT device. The difference between those results could be explained by improved distinction of BLamDs and BM due to superior axial resolution. Thus, the High-Res OCT device can be beneficial in predicting future growth of atrophy, as BLamDs, corresponding to the thin double-layer sign in OCT, are a risk factor for conversion to cRORA.^47^^,^^48^

One difficulty associated with quantification of the outer retinal bands in GA is the differentiation of drusen, SDDs, and pigmentary migrations such as sloughed (Fig. 5C), shedding (Fig. 5B), or bilaminar (Fig. 5D) RPE. The High-Res OCT provides a clear image of the outer retinal bands, as well as structural patterns, such as SDDs and different RPE phenotypes. Detailed visualization revealed small hyperreflective circles located between the inner boundary of the IZ and the outer boundary of the EZ bands without hyporeflective tails (Fig. 5A). In this study, we have referred to this structure as hyperreflective spots. These hyperreflective spots could derive from sloughed RPE (Fig. 5C), which has been described in histologic studies as spherical RPE cells in the subretinal space with intact underlying epithelium.^21^ Therefore, we suggest these subretinal hyperreflective dots could be apically sloughed RPE or shedded granule aggregates from the underlying RPE cells. Another hypothesis would be phagocytized RPE by monocyte-derived macrophages retaining melanosomes.^21^ However, more studies correlating histologic phenotypes of RPE to high-resolution OCT imaging are necessary to confirm these theories.

Our results showed decreased SDD volumes for the central subfield (all *P* < 0.001) with the highest volumes in the superior quadrant being 29.8 nL. These differences may not be significant due to the small sample size in this study (all *P* < 0.05) (Fig. 4). In accordance with our findings, previous research evaluating the topographic prevalence of SDDs in non-neovascular AMD reported that SDDs rarely accumulated in the fovea (7.7%–9.9%).^49^ Although the inferior subfield was not sectioned, that study reported the highest prevalence of SDDs in the superior perifovea (62%) followed by the nasal (17.5%) and temporal (10.5%) perifovea.^49^ Another study reported a higher amount of SDDs in the superior hemifield compared to inferior, whereas no SDDs were found in the central circle of all eyes.^50^ Furthermore, Saßmannshausen et al.^51^ evaluated the topographic distribution of SDDs by using near-infrared reflectance, green and blue fundus autofluorescence, and retro mode imaging. Similarly, they found numerous SDDs in the outer ETDRS rings, especially superiorly, and fewer SDDs in the fovea. All publications have concluded that SDDs are more prevalent outside the foveal center following the distribution of rod PRs,^49^^–^^52^ whereas high rod density has been found particularly superior or superior–temporal to the fovea.^53^ Therefore, our results confirm a lower prevalence of SDDs in the central 1-mm area, whereas the distribution of SDDs outside of the foveal center would have to be verified in further research with a larger sample size using multimodal imaging anchored by OCT which could improve distinguishing between SDDs and nonuniform RPE phenotypes.

The lack of precise definitions of these structures for OCT-based quantification limits the reliability of outer retinal band quantifications to a certain degree. Inter-reader agreement reported relatively high subjectivity for assessing the presence and number of SDDs, particularly for stage 1 SDDs, which were defined as any diffuse hyperreflective material between the RPE and the EZ bands. Additionally, in the High-Res OCT, two lines presumably corresponding to ROSTs and COSTs became visible, which could be mistaken for stage 1 SDDs. To minimize this limitation, stage 1 SDDs were not segmented in this study.^24^ Despite substantial or near-substantial agreement on the presence of SDDs in previous research, merely fair inter-reader agreement was found for evaluating the number of SDDs on OCT B-scans for standard SPECTRALIS HRA+OCT. The authors concluded that the manual quantification of SDDs in standard OCT devices would be more variable, highlighting the need for superior resolution imaging, as SDDs are an important risk factor for the progression of GA.^24^ Previous assessments of intra-reader variability in the High-Res OCT reported mean absolute errors ranging from 1.7 for ELM segmentation to 3.5 for segmentation of the RPE. In addition to inter-reader variability (mean absolute errors of 3.1 for ELM and 5.9 for RPE), these data show that, despite superior axial resolution, a small variability remains in manual corrections of layer segmentations.^15^ Nevertheless, two studies reported superior agreement in the High-Res OCT compared to the standard SPECTRALIS, underlining the benefits of the High-Res OCT.^15^^,^^16^ One strength of this study is a reader manual containing precise definitions, which readers received prior to manual corrections next to regular training in the differentiation of these characteristic changes. Further strengths of this study include follow-up acquisitions with manual fovea annotations, a substantial number of B-scans analyzed, and manually corrected topographic measurements of GA-relevant retinal features in two devices. Clinicians may use these quantifications when examining the natural history of GA as a reference for patient management, as novel therapies demand a selection of suitable candidates for treatment with complement inhibitors. However, further studies are necessary to confirm these benefits. Furthermore, the lack of longitudinal data and the relatively small sample size could explain similar results in RPE loss, limiting the validity of this study.

In conclusion, to the best of our knowledge, this study represents the first assessment of differences between superior and standard axial resolution OCT devices via AI-assisted layer segmentation in eyes with GA. Significant differences between the devices were found regarding outer retinal band thickness and integrity loss in GA, highlighting the potential of High-Res OCT imaging. Combined with recent milestones achieved in GA therapy, the High-Res OCT device could be of benefit in patient selection and evaluating therapeutic efficacy in the future.
